# Retraction: Overexpression of the Insulin Receptor Isoform A Promotes Endometrial Carcinoma Cell Growth

**DOI:** 10.1371/journal.pone.0260686

**Published:** 2021-11-23

**Authors:** The PLOS ONE Editors

Following the publication of this article [1], concerns were raised regarding results presented in Figs 2, 6, and 7. Specifically,

- The following results appear more similar than would be expected from independent samples:
      ○ Fig 2A, IR-B/IR-A panel lanes T10 and T15.
      ○ Fig 2A, IR-B/IR-A panel lanes T11 and T16, and T18.
      ○ Fig 2A, IR-B/IR-A panel lanes T17 and T19.
      ○ Fig 2A, GAPDH panel lanes T10-T11 and lanes T12-T13 respectively.
      ○ Fig 2A, GAPDH panel lanes T14-T16 and lanes T17-T19 respectively.
      ○ Fig 2B, IR-B/IR-A panel lanes N16 and N17.
      ○ Fig 2B, GAPDH panel lanes N10-N14 and lanes N15-N19 respectively.
- The IR-B/IR-A panel of Fig 2B appears to have been spliced between N10-N11, N12-N13, N13-N14, and N14-N15. The corresponding GAPDH blot does not appear to have been spliced in the same regions.
- Fig 6 and Fig 7 report the same results. The submission files indicated that this was likely due to a production error. The authors noted that Fig 6 was published correctly and that the wrong image was published as Fig 7.

The authors clarify that the blots presented in Fig 2 were cut and spliced for the purpose of figure beautification. The original data underlying the published results are no longer available. Following discussion of these concerns, the corresponding author requested withdrawal of the article.

In light of the unresolved concerns about Fig 2, the *PLOS ONE* Editors retract this article.

All authors agreed with the retraction. CFW, GZ, LJZ, WJQ, and LHW also stated that they stand by the article’s findings and apologize for the issues with the published article.
